# Fluid Rate Is Important As Much As Fluid Tonicity: An Experimental Study

**DOI:** 10.5152/eurasianjmed.2021.20276

**Published:** 2021-06

**Authors:** Halil Keskin, Filiz Keskin, Zuhal Keskin Yildirim, Muhammet Akif Guler, Nurinnisa Ozturk, Berna Ozturk Karagoz, Zekai Halici

**Affiliations:** 1 Division of Pediatric Intensive Care Unit, Department of Pediatrics, Atatürk University Faculty of Medicine, Erzurum, Turkey; 2 Clinical Research, Development and Design Application and Research Center, Atatürk University, Erzurum, Turkey; 3 Division of Pediatric Neurology, Department of Pediatrics, Atatürk University Faculty of Medicine, Erzurum, Turkey; 4 Division of Pediatric Oncology, Department of Pediatrics, Atatürk University Faculty of Medicine, Erzurum, Turkey; 5 Department of Pediatrics, Atatürk University Faculty of Medicine, Erzurum, Turkey; 6 Department of Biochemistry, Atatürk University Faculty of Medicine, Erzurum, Turkey; 7 Department of Pharmacology, Atatürk University Faculty of Medicine, Erzurum, Turkey; 8 Department of Pharmacology, Atatürk University Faculty of Medicine, Erzurum, Turkey

**Keywords:** Different infusion rate, fluid tonicity, maintenance intravenous fluids, plasma osmolality, rat

## Abstract

**Objective:**

There is no study evaluating the effect on plasma osmolality of both fluid tonicity and high fluid rate at the same time. The aim of this experimental study was to determine the change in the plasma osmolality by different fluid tonicity and rate, and to suggest the safest and the most appropriate fluids based on the plasma osmolality for medical situations requiring fluid therapy with high or maintenance rates.

**Materials and methods:**

The rats were randomly divided into seven groups (six rats in each group): [D_5_] D_5_ administered at 100 ml/kg/24h; [D_5_150] D_5_ administered at 150 ml/kg/24h; [D_5_(½)100] D_5_ 0.45% NaCl administered at 100 ml/kg/24h; [D_5_(½)150] D_5_ 0.45% NaCl administered at 150 ml/kg/24h; [D_5_(1)100] D_5_ 0.9% NaCl administered at 100 ml/kg/24h; [D_5_(1)150] D5 0.9% NaCl administered at 150 ml/kg/24h; [Control group] non-treated control rats. Intracardiac blood samples were collected from all the groups at the end of 24 h.

**Results:**

[D_5_(1)150] and [D5(½)100] were the group closest to the control group in terms of both sodium (*P* = .937; *P* = .699, respectively) and effective osmolality (*P* = 1, *P* = .818, respectively).

**Conclusion:**

Our results showed that 0.9% NaCl and 0.45% NaCl solutions might be the safest and the most appropriate fluids to maintain normal plasma osmolality in medical situations requiring fluid therapy with high or maintenance rates, respectively.

## Introduction

All intravenous fluids (IVFs) having a sodium content < 0.9% NaCl are regarded as hypotonic. Although the use of hypotonic IVFs has become standard practice^1^, many studies have shown that the use of hypotonic IVFs causes hyponatremia.^2^–^5^ Those studies have suggested that isotonic rather than hypotonic IVFs should be used. The American Academy of Pediatrics also strongly recommends the use of isotonic IVFs.^1^ However, the tonicity of IVFs is not the only determinant to prevent hyponatremia. The fluid rate or amount of fluid is another key factor, and it also should be taken into consideration when IVFs are chosen. The maintenance rate or amount of maintenance fluid is determined based on the Holliday and Segar method.^6^ Accordingly, the high rate or high amount of fluid is based on the determination made by that method. Maintenance IVF therapy and associated hyponatremia remain an important topic, and studies on the subject are ongoing.^7^–^10^ In the literature, there is no study evaluating the effect on plasma osmolality of both fluid tonicity and high fluid rate at the same time, although previous studies have shown the effect on plasma osmolality based on the maintenance or low rate alone.^11^–^13^ The aim of this experimental study was to determine the change in plasma osmolality due to different fluid tonicity and rate and to suggest the safest and the most appropriate fluids based on plasma osmolality for medical conditions requiring fluid therapy with high or maintenance rates.

## Materials and Methods

### Animals

In total, 42 male albino Wistar rats weighing 250–280 g were used in the experiments. The animals were obtained from Ataturk University’s Experimental Animal Laboratory at the Medicinal and Experimental Application and Research Centre. The animal experiments and procedures were performed in accordance with national guidelines for the use and care of laboratory animals and approved by Ataturk University’s local animal care committee (2019/88).

The rats were housed on sawdust bedding in standard plastic cages in a well-ventilated room at 22 °C under specific light conditions (a 14:10 hour light: dark cycle). The animals were fed a standard diet and had access to tap water ad libitum. The rats were not fasted before the experiment and were randomly divided into 7 groups, with 6 animals in each group. The weights of the rats were recorded before the experiment (Table 1).

### Experimental Design

The treatment groups were as follows:

- D_5_100, administered dextrose 5% at 100 mL/kg/day by continuous intravenous infusion
- D_5_150, administered dextrose 5% at 150 mL/kg/day by continuous intravenous infusion
- D_5_(½)100, administered dextrose 5% and NaCl 0.45% at 100 mL/kg/day by continuous intravenous infusion
- D_5_(½)150, administered dextrose 5% and NaCl 0.45% at 150 mL/kg/day by continuous intravenous infusion
- D_5_(1)100, administered dextrose 5% and NaCl 0.9% at 100 mL/kg/day by continuous intravenous infusion
- D_5_(1)150, administered dextrose 5% and NaCl 0.9% at 150 mL/kg/day by continuous intravenous infusion
- Control group, no administration of any fluid

Intraperitoneal thiopental (25–35 mg/kg) and ketamine (25–35 mg/kg) were administered to the rats for anesthesia. The tail was first immersed in warm water (40°C) to make the tail vein of the anesthetized animals visible. The tail was then cleaned with alcohol. Vascular access was achieved using a 26 gauge intravenous needle and fixed with a tongue depressor rod. The rats were infused with midazolam (0.5–0.7 mg/kg/hour) for 24 hours with the aid of an infusion pump.

In the experiment, 3 different fluids (dextrose 5%, dextrose 5% and NaCl 0.45%, or dextrose 5% and NaCl 0.9%) were administered at different delivery rates (100 mL/kg/day or 150 mL/kg/day) for 24 hours, depending on the group. Intracardiac blood samples were collected from all groups at the end of 24 hours.

The animals were scarified by high-dose anesthesia. Blood samples were obtained for analyses of serum levels of sodium (Na^+^), chloride (Cl^−^), and glucose and analyses of pH values. Effective serum osmolality was then calculated. All analyses were conducted by staff in the biochemistry department of the Facility of Medicine.

### Sample Collection and Biochemical Measurements

Whole-blood samples collected with blood gas syringes containing heparin (SafePico, Radiometer, Copenhagen, Denmark) were analyzed in a point-of-care blood gas analyzer (ABL800 FLEX, Radiometer) to determine pH and glucose, Na^+^, and Cl^−^ concentrations. The effective serum osmolality value was calculated using the following formula: [(2 × Na^+^) + (Glu/18)]. The biochemical data are presented in Table 1.

### Statistical Analysis

The fit of the data to a normal distribution was determined by analytical and graphical methods. Parametric tests were performed for data that complied with a normal distribution, and nonparametric tests were performed for data that did not comply with a normal distribution. When the data were analyzed, only glucose levels had a normal distribution. Thus, the glucose data were compared with Duncan’s post hoc multiple comparison test in a one-way analysis of variance test. The other parameters were analyzed using the Kruskal-Wallis test. In addition to the Na^+^ and Cl^−^ concentrations and effective serum osmolality data, in which the difference was significant, the Mann-Whitney *U* test was used to compare the groups two by two. All statistical calculations were performed using the Statistical Package for Social Sciences software version 20.0 (IBM SPSS Corp.; Armonk, NY, USA) statistical program. *P*-values ≤ .05 were considered significant.

### Ethics Committee Approval

The study was approved by the local animal care committee of Ataturk University (April 30^th^, 2019; Meeting no: 5; Decree no: 88).

## Results

There was no significant difference in the glucose levels, pH values, and rat weights in each group versus those in all other groups (*P* > .05).

### Analysis of Sodium Levels (Table 2)

As compared with the level in the control group, the lowest Na^+^ level was detected in the D_5_150 group, which received only dextrose at 150 mL/kg/d. The decrease in the Na^+^ level in the D_5_150 group was significantly lower than that in all other groups (*P* < .05) except the D_5_100 group (*P* > .05).

Na^+^ levels in the D_5_100 group were significantly lower than all other groups (*P <* .05) except the D_5_150 and D_5_(½)150 groups (*P* > .05).

Na^+^ levels in the D_5_(½)100 group were significantly higher than those in the D_5_100 and D_5_150 groups (*P* < .05) and significantly lower than those in the D_5_(1)100 group (*P* < .05). Na^+^ levels in the D_5_(½)100 group were also higher than those of the D_5_(½)150 group that received the same fluid at a different rate. There was no statistically significant difference between the Na^+^ levels of the D_5_(1)150 group and those in the control group (*P* > .05).

Similarly, in the D_5_(½)150 group, Na^+^ levels were significantly decreased when compared with the control, D_5_(½)100, D_5_(1)100, and D_5_(1)150 groups (*P* < .05). The Na^+^ level of the D_5_(½)150 group was similar to that of the D_5_100 group, which received no Na^+^ (*P* > .05). The Na^+^ depletion that occurred in the D_5_(½)150 group was not as critical as in the D_5_150 group.

There was no statistically significant difference between the Na^+^ levels of the D_5_(1)100 group and those of the control group (*P* > .05), but the levels in the D_5_(1)100 group were higher than those of the other groups (*P* < .05).

The Na^+^ levels in the D_5_(1)150 group were closest to those in the control group (*P* = .937). An interesting finding in this group was the lack of statistical difference between the control group and the D_5_(½)100 group (*P* = .699).

The D_5_(½)100, D_5_(1)100, D_5_(1)150, and control groups were characterized by isonatremia, and the D_5_100, D_5_150, and D_5_(½)150 groups were characterized by hyponatremia. Hypernatremia was not detected in any group.

### Analysis of Chloride Levels (Table 3)

As compared with the control group, the Cl^−^ levels in the D_5_150 group (did not receive Cl^−^ and received IVF at a high rate) were the lowest, similar to the Na^+^ levels. There was no statistically significant difference in the Cl^−^ levels in the D_5_150 and D_5_100 groups (*P* > .05), whereas there was a significant decrease in the levels in the D_5_150 group as compared with those in the other groups (*P* < .05).

The Cl^−^ levels in the D_5_100 group were significantly lower than all other groups (*P* < .05) except the D_5_150 group (*P* > .05).

The Cl^−^ levels in the D_5_(½)100 group were significantly higher than those in the D_5_100 and D_5_150 groups (*P* < .05), and they were significantly lower than those in the D_5_(1)100 group (*P* < .05). There was no statistically significant difference between the Cl^−^ levels of the D_5_(½)100 group and those in the D_5_(½)150, D_5_(1)150, and control groups (*P* > .05).

The Cl^−^ levels in the D_5_(½)150 group were significantly higher than those in the D_5_100 and D_5_150 groups (*P* < .05) and significantly lower than those in the D_5_(1)100 group (*P* < .05). However, there was no statistically significant difference between the Cl^−^ levels of the D_5_(½)150 group and the D_5_(½)100, D_5_(1)150, and control groups (*P* > .05).

There was also no statistically significant difference between the Cl^−^ levels in the D_5_(1)100 group and those in the D_5_(1)150 and control groups (*P* > .05). However, the Cl^−^ levels in the D_5_(1)100 group were higher than those in the other groups (*P* < .05).

In line with the findings for Na^+^ levels, the D_5_(1)150 group was the closest group to the control group in terms of Cl^−^ levels (*P* = .818). The highest Cl^−^ level was found in the D_5_(1)100 group. This group also had the highest Na^+^ level.

### Analysis of Effective Osmolality Values (Table 4)

As compared with the values in the control group, the effective serum osmolality values were lowest in the D_5_150 group (not containing Na^+^ and a high rate of delivery). There was no statistically significant difference in the effective serum osmolality values of the D_5_150 and D_5_100 groups (*P* > .05), but the values in the D_5_150 group were significantly decreased compared with those of the other groups (*P* < .05).

There was no significant difference between the effective serum osmolality values of the D_5_100 group and those of the D_5_150 and D_5_(½)150 groups (*P* > .05). There was a statistically significant decrease in the effective serum osmolality values as compared with those of the other groups (*P <* .05).

The effective serum osmolality levels in the D_5_(½)100 group were significantly higher than those in the D_5_100 and D_5_150 groups (*P* < .05) and significantly lower than those in the D_5_(1)100 group (*P* < .05). Effective serum osmolality levels in the D_5_(½)100 group were also higher than those of the D_5_(½)150 group that received the same fluid at a different rate. There was no statistically significant difference between the effective serum osmolality levels of the D_5_(1)150 group and those in the control group (*P* > .05).

As in the D_5_100 group, in the D_5_(½)150 group, effective serum osmolality levels were significantly decreased when compared with the control, D_5_(1)100, and D_5_(1)150 groups (*P* < .05). The effective serum osmolality level of the D_5_(½)150 group was similar to that of the D_5_100 group, which received no Na^+^ (*P* > .05). The effective serum osmolality depletion that occurred in the D_5_(½)150 group was not as critical as in the D_5_150 group.

Although there was no statistically significant difference between Na^+^ levels in the D_5_(1)100 group and those in the control group, the effective serum osmolality values of the D_5_(1)100 group were higher than those of the other groups (*P* < .05).

The effective serum osmolality values of the D_5_(1)150 group were closest to those of the control group (*P* = 1). There was no statistically significant difference between the effective serum osmolality values of the D_5_(½)100 group and those of the control group (*P* = .818). The effective serum osmolality values of the D_5_(½)100 group were significantly lower than those of the D_5_(1)100 group and significantly higher than those of the D_5_100, D_5_150, and D_5_(½)150 groups (*P* < .05).

## Discussion

Maintenance IVF therapy, common in the pediatric population, has a lot of differences in terms of fluid content because there are no guidelines on the components of IVFs or the electrolyte contents.^14^–^17^ IVF therapy should be identified by the medical situation.^18^,^19^ Some patients only need maintenance fluid, whereas others having a medical condition, such as tumor lysis syndrome, or intoxication need a high amount of fluid. The primary goal in IVF therapy is to keep plasma osmolality in normal limits. Fluid rate may also be important as much as its content for this reason. In this context, to the best of our knowledge, this research is the first study evaluating the effect on plasma osmolality of both fluid tonicity and high fluid rate at the same time. This study shows that the fluid rate should be taken into consideration while deciding on IVFs.

In this study, it was not surprising that D_5_100 and D_5_150 caused hyponatremia, hypochloremia, and low plasma osmolality. As glucose rapidly penetrates cells, it only has a limited effect on the plasma osmolality.^1^ In such a case, the free water in the intravascular area results in low plasma osmolality. Surprisingly, the D_5_(½)150 group also had hyponatremia and low plasma osmolality. This fluid containing 77 mEq/L of Na^+^ is often preferred in IVF therapy.^20^ Our finding suggests how important fluid rate also is to prevent hyponatremia. Previous studies and textbooks usually suggest the use of hypotonic IVFs because isotonic IVFs are linked to hypernatremia, hyperchloremic metabolic acidosis, edema, and hypertension.^21^,^22^ However, those studies were just focused on fluid tonicity, not fluid rate. Additionally, in this study, D_5_(1)100, but not D_5_(1)150, had hypernatremia, acidosis, and hyperosmolality. This finding shows us that the fluid rate is important to prevent not only hyponatremia but also hypernatremia.

The most remarkable finding of this study was the similarity of measured biochemical markers in the D_5_(1)150 and control groups. Our results clearly demonstrate that isotonic fluids should be preferred, especially in medical situations requiring a high fluid rate. Additionally, the fluid containing 77 mEq/L of Na^+^ (dextrose 5%, NaCl 0.45%) should be chosen only when it is given at the maintenance rate.

A limitation of this experimental study was that the rats were healthy. In case of an acute disease, a dilutional hyponatremia develops as a result of free water retention in the body owing to the increased arginine vasopressin effect.^20^ In such cases, the fluid tonicity and rate should be arranged by the strict monitorization of clinical findings, urinary output, and biochemical markers.

In conclusion, this is the first study evaluating the effect on plasma osmolality of both fluid tonicity and high rate at the same time. Our results showed that NaCl 0.9% and NaCl 0.45% solutions might be the safest and the most appropriate fluids to maintain normal plasma osmolality in medical situations requiring fluid therapy with high or maintenance rates, respectively. However, new studies showing the effect on plasma osmolality of both fluid tonicity and rate on the patients are needed.

**Main Points**

- In children who cannot obtain sufficient fluid via enteral feeding for various reasons, maintenance intravenous fluids (IVFs) are to be used.
- During fluid therapy in pediatrics, the same protocol cannot be applied to all patients, because the fluid content of each patient needs to be adjusted according to the needs of the individual patient.
- There is no study evaluating the effect on plasma osmolality of both fluid tonicity and high fluid rate at the same time.
- We can suggest that among patients who required larger amounts of IVFs than determined by the Holliday and Segar calculation, the type of selected fluid will be quite important in terms of the electrolyte balance.

## Figures and Tables

**Table 1:** Data from Rats

Variable	n	Weight (g)	Na+ (mEq/L)	Cl^−^ (mEq/L)	Glu (mg/dL)	pH	EfOsm (mOsm/L)
D_5_100	6	260 ± 24.2	127.5 ± 3.3	103.5 ± 2.1	149.5 ± 9.2	7.39 ± 0.3	263.5 ± 6.3
D_5_150	6	260 ± 23.67	124 ± 5.6	99 ± 5.7	151.5 ± 10.6	7.39 ± 0.2	256.1 ± 10.9
D_5_(½)100	6	260 ± 31.9	139.5 ± 1.5	111.5 ± 1.9	150.5 ± 4.9	7.36 ± 0.3	287.4 ± 3.2
D_5_(½)150	6	265 ± 26.1	130.5 ± 3.3	110.5 ± 3.3	150.5 ± 7.7	7.38 ± 0.4	269.3 ± 6.3
D_5_(1)100	6	265 ± 27.9	142.5 ± 2	115.5 ± 1.4	147.5 ± 3.4	7.37 ± 0.3	293.4 ± 4
D_5_(1)150	6	265 ± 22.5	139.5 ± 1.6	114 ± 2	146.5 ± 8.8	7.38 ± 0.3	287.2 ± 2.9
Control	6	265 ± 19.4	139.5 ± 2.3	112.5 ± 3.5	148.5 ± 8.7	7.37 ± 0.3	286.9 ± 4.4

Results are shown as median ± SD.

D_5_100: 100 mL/kg/d of D_5_; D_5_150: 150 mL/kg/d of D_5_; D5(½)100: 100 mL/kg/d of D_5_ and 0.45% NaCl; D_5_(½)150: 150 mL/kg/d of D_5_ and 0.45% NaCl; D_5_(1)100: 100 mL/kg/d of D_5_ and 0.9% NaCl; D_5_(1)150: 150 mL/kg/d of D_5_ and 0.9% NaCl; Control: no fluid.

Cl^−^, chloride; D_5_, dextrose 5%; EfOsm, effective osmolality; Glu, glucose; Na+, sodium; SD, standard deviation.

**Table 2:** Sodium Values (MEq/L) and Comparison Between All Groups

Variable	Median ± SD	*P*-Value					

		D_5_150	D_5_(½)100	D_5_(½)150	D_5_(1)100	D_5_(1)150	Control
D_5_100	127.5 ± 3.3	.240	.002	.093	.002	.002	.002
D_5_150	124.0 ± 5.6	—	.002	.041	.002	.002	.002
D_5_(½)100	139.5 ± 1.5	—	—	.002	.009	.699	.699
D_5_(½)150	130.5 ± 3.3	—	—	—	.002	.002	.002
D_5_(1)100	142.5 ± 2.0	—	—	—	—	.015	.065
D_5_(1)150	139.5 ± 1.6	—	—	—	—	—	.937
Control	139.5 ± 2.3	—	—	—	—	—	1

Kruskal-Wallis test was used for comparison between all groups. Because the difference was significant according to the Kruskal-Wallis test (P < .05), additional Mann-Whitney U tests were used for binary group comparisons. P-values of Mann-Whitney U test are represented in intersections of the groups.

Results are shown as median ± SD.

D_5_100: 100 mL/kg/d of D_5_; D_5_150: 150 mL/kg/d of D_5_; D_5_(½)100: 100 mL/kg/d of D_5_ and 0.45% NaCl; D_5_(½)150: 150 mL/kg/d of D_5_ and 0.45% NaCl; D_5_(1)100: 100 mL/kg/d of D_5_ and 0.9% NaCl; D_5_(1)150: 150 mL/kg/d of D_5_ and 0.9% NaCl; Control: no fluid.

D_5_, dextrose 5%; SD, standard deviation.

**Table 3:** Chloride Values (mEq/L) and Comparison Between All Groups

Variable	Median ± SD	*P*-Value					

		D_5_150	D_5_(½)100	D_5_(½)150	D_5_(1)100	D_5_(1)150	Control
D_5_100	103.5 ± 2.1	.065	.002	.004	.002	.002	.002
D_5_150	99.0 ± 5.7	—	.004	.009	.002	.002	.004
D_5_(½)100	111.5 ± 1.9	—	—	.589	.004	.065	.394
D_5_(½)150	110.5 ± 3.3	—	—	—	.009	.065	.180
D_5_(1)100	115.5 ± 1.4	—	—	—	—	.240	.394
D_5_(1)150	114.0 ± 2.0	—	—	—	—	—	.818
Control	112.5 ± 3.5	—	—	—	—	—	1

Kruskal-Wallis test was used for comparison between all groups. Because the difference was significant according to the Kruskal-Wallis test (*P* < .05), additional Mann-Whitney U tests were used for binary group comparisons. P-values of Mann-Whitney U test are represented in intersections of the groups.

Results are shown as median ± SD.

D_5_100: 100 mL/kg/d of D_5_; D_5_150: 150 mL/kg/d of D_5_; D_5_(½)100: 100 mL/kg/d of D_5_ and 0.45% NaCl; D_5_(½)150: 150 mL/kg/d of D_5_ and 0.45% NaCl; D_5_(1)100: 100 mL/kg/d of D_5_ and 0.9% NaCl; D_5_(1)150: 150 mL/kg/d of D_5_ and 0.9% NaCl; Control: no fluid.

D_5_, dextrose 5%; SD, standard deviation.

**Table 4:** Effective Serum Osmolality Values (mOsm/L) and Comparison Between All Groups

Variable	Median ± SD	*P*-Value					

		D_5_150	D_5_(½)100	D_5_(½)150	D_5_(1)100	D_5_(1)150	Control
D_5_100	263.5 ± 6.3	0.180	0.002	0.130	0.002	0.002	0.002
D_5_150	256.1 ± 10.9	—	0.002	0.041	0.002	0.002	0.002
D_5_(½)100	287.4 ± 3.2	—	—	0.002	0.015	0.699	0.818
D_5_(½)150	269.3 ± 6.3	—	—	—	0.002	0.002	0.002
D_5_(1)100	293.4 ± 4.0	—	—	—	—	0.009	0.041
D_5_(1)150	287.2 ± 2.9	—	—	—	—	—	1
Control	286.9 ± 4.4	—	—	—	—	—	1

Kruskal-Wallis test was used for comparison between all groups. Because the difference was significant according to the Kruskal-Wallis test (P < .05), additional Mann-Whitney U tests were used for binary group comparisons. P-values of Mann-Whitney U test are represented in intersections of the groups.

Results are shown as median ± SD.

D_5_100: 100 mL/kg/d of D_5_; D_5_150: 150 mL/kg/d of D_5_; D_5_(½)100: 100 mL/kg/d of D_5_ and 0.45% NaCl; D_5_(½)150: 150 mL/kg/d of D_5_ and 0.45% NaCl; D_5_(1)100: 100 mL/kg/d of D_5_ and 0.9% NaCl; D_5_(1)150: 150 mL/kg/d of D_5_ and 0.9% NaCl; Control: no fluid.

D_5_, dextrose 5%; SD, standard deviation.
